# Emotional intelligence weakly predicts academic success in medical programs: a multilevel meta-analysis and systematic review

**DOI:** 10.1186/s12909-023-04417-8

**Published:** 2023-06-08

**Authors:** Ahmed M. Abdulla Alabbasi, Fatema A. Alabbasi, Aseel AlSaleh, Ahmed M. Alansari, Reginald P. Sequeira

**Affiliations:** 1 Department of Gifted Education, Arabian Gulf University, P.O. Box: 26671, Manama, Kingdom of Bahrain; 2 College of Medicine and Medical Sciences, Arabian Gulf University, Manama, Bahrain; 3 Department of Family and Community Medicine, Arabian Gulf University, Manama, Bahrain; 4 Government Hospitals, Manama, Kingdom of Bahrain; 5 Department of Pharmacology and Therapeutics, Arabian Gulf University, Manama, Bahrain

**Keywords:** Emotional intelligence, Undergraduate medical students, Academic success, Meta-analysis, Doctor of medicine

## Abstract

**Background:**

Emotional intelligence (EI) is a predictive factor of academic success in undergraduate Doctor of Medicine (MD) programs. Although some research suggests a positive association between EI and academic success in MD programs, other research reports neither an association nor a negative correlation between the two variables. The current study aimed to resolve these contradictory findings by conducting a systematic review and a meta-analysis using research from 2005 to 2022.

**Methods:**

Data were analyzed using a multilevel modeling approach to (a) estimate the overall relationship between EI and academic success in MD programs and (b) determine whether the mean effect size varies according to country (United States vs. non-United States countries), age, EI test, EI task nature (ability-based vs. trait-based), EI subscales, and academic performance criteria (grade point average vs. examinations).

**Results:**

Findings from 20 studies (*m* = 105; *N* = 4,227) indicated a positive correlation between EI and academic success (*r* = .13, 95% CI [.08, – .27], *p* < .01). Moderator analyses indicated that the mean effect size significantly varied according to EI tests and EI subscales. Moreover, three-level multiple regression analyses showed that between-study variance explained 29.5% of the variability in the mean effect size, whereas within-study variance explained 33.5% of the variability in the mean effect.

**Conclusions:**

Overall, the current findings show that EI is significantly, albeit weakly, related to academic success in MD programs. Medical researchers and practitioners can therefore focus on integrating EI-related skills into the MD curriculum or target them through professional development training and programs.

## Background

Which factors predict academic success in undergraduate medical programs? While some students perform well in Doctor of Medicine (MD) programs, others fail to complete their studies or struggle in their MD journey. Moreover, the current competencies and expectations of undergraduate medical students differ from those of the last 20 to 30 years [1], which highlights the significance of revising the admission criteria for such programs [2]. Numerous research studies in medical education have evaluated the admission criteria as determinants of academic success for MD students. Over the last two decades, medical education researchers have attempted to test the association between academic success and factors, such as metacognitive awareness [3, 4], motivation [5*, 6, 7], coping strategy [8, 9], learning style [10, 11], educational environment [12], critical thinking [13, 14], and Emotional Intelligence (EI) [15*, 16*, 17]. In this study, we have comprehensively investigated EI as the variable of interest.

Although the notion of EI was first discussed by Edward Thorndike in the 1920s when he conceptualized intelligence as a multidimensional rather than unidimensional construct, including mechanical, abstract, and *social intelligence*, it was Salovey and Mayer’s seminal work that contributed to the systematic and scientific study of EI [18]. Since then, other theories and models of EI such as Bar-On’s model [19] and Goleman’s model [20] have been introduced. EI has been extensively researched in different fields including sports, education, music, and medicine [21–24]. It is increasingly becoming important in the medical profession because success in this field is not only determined by knowledge and academic excellence but also the acquisition of EI-related skills such as empathy, communication, interpersonal sensitivity, and emotion recognition [16*]. Doctors manage different kinds of patients with varying socioeconomic status, and diverse case severity that range from mild to critical illnesses, which requires an understanding of patients’ emotions, ability to demonstrate empathy, and in some instances, communicate bad news in a professional way.

Interest in studying the association between EI and academic success in MD programs arose in the first decade of the twenty-first century. Notably, a literature review on the predictors of academic success in medical schools revealed that EI is one of the most studied variables, with three systematic reviews published on this topic [17, 25, 26]. One of the major findings of these systematic reviews was that primary studies reported contradictory findings. For instance, Singh, Kulkarni, and Gupta [17] reported that eight studies concluded that EI has a positive impact on academic success, two studies showed nonsignificant associations between EI and academic success, and 11 studies showed a negative relationship between EI and academic success. A similar conclusion was reached by Arora et al. [25] and Cook, Cook, and Hilton [26]. Although systematic reviews offer valuable source information for researchers regarding the effectiveness of an intervention, the difference between two or more groups for a specific variable, and the association between different factors, they do not provide quantitative and reliable results. Therefore, one of the objectives of the current study is to synthesize the effect sizes stemming from primary studies using a multilevel meta-analysis approach to clarify the nature and magnitude of the relationship between EI and academic success in MD programs. The second objective is to identify factors that may contribute to the contradictory findings in primary studies (see Table 1).

**Table 1:** Included studies on the relationship between EI and academic success

Authors	Location	EI test	Task nature	Academic performance criterion	Major findings
Austin et al. [23*]	United Kingdom	EI questionnaire	Trait-based EI	• Unit examination	Examination performance was positively and significantly related to the EI score
Stratton et al. [27*]	United States	Trait Meta Mood Scale (TMMS)	Trait-based EI	• Comprehensive clinical performance (CPX) • Physical examination (PE)	A positive significant correlation was found with the Attention to Feelings subscale of the TMMS PE, while other TMMS subscales were not significantly correlated with either CPX or PE
Austin et al. [28*]	United Kingdom	SSEIT	Trait-based EI	• End-of-year examination	Overall, no association between EI and academic performance was found
Carr [29*]	Australia	MSCEIT	Ability-based EI	• Undergraduate Medicine and Health Sciences Admission Test (UMAT) • Tertiary Entrance Rank (TER) scores	No significant correlations were found between EI and the parameters of academic performance
Fallahzadeh [30*]	Iran	EQ-i	Trait-based EI	• GPA	A positive significant relationship was found between total EI, the Stress Management subscale, the Adaptability subscale, and academic performance
Leddy et al. [31*]	Canada	MSCEIT	Ability-based EI	• Weighted GPA	A negative significant correlation was found between EI and wGPA
Chew, Zain, and Hassan [32*]	Malaysia	MSCEIT	Ability-based EI	• Total continuous assessment • Final examination marks	A positive significant correlation was observed between total EI and both total continuous assessment and final examination marks
Brannick et al. [33*]	United States	Wong and Law Emotional Intelligence Scale (WLEIS) MSCEIT	Trait-based EI Ability-based EI	• Medical College Admissions Test (MCAT) • GPA	There was no significant correlation between trait-based EI (i.e., WLEIS) and academic performance, while ability-based EI (i.e., MSCEIT) was significantly correlated with academic performance
Shah et al. [34*]	India	EQ questionnaire prepared by the Institute of Health and Human Potential	Trait-based EI	• Achievement examination	A significant negative correlation was found between EI and academic achievement
Humphrey-Murto et al. [15*]	Canada	MSCEIT	Ability-based EI	• Written examination • Objective structured clinical examination	EI does not appear to reliably predict future academic performance
Naeem et al. [35*]	Saudi Arabia	SSEIT	Ability-based EI	• Cumulative grade point average (CGPA)	A significant positive relationship was found between EI and the Optimism subscale, and EI and the Awareness of Emotions subscale, while EI was not significantly associated with the Use of Emotion subscale
Libbrecht et al. [16*]	Belgium	Situational Test of Emotional Understanding (STEU) Situational Test of Emotion Management (STEM)	Ability-based EI	• Standardized grades assessing two facets of academic performance: (a) intellectual academic performance, and (b) interpersonal academic performance	A significant positive correlation was observed between the Emotional Regulation Ability subscale and total EI and Interpersonal Academic Performance, while no significant association was found between EI and Intellectual Academic Performance
Radfar et al. [36*]	Iran	EQ-i	Trait-based EI	• GPA	There was a positive significant relationship between EI and GPA
Rajasingam et al. [37*]	Malaysia	Trait Meta Mood Scale (TMMS)	Trait-based EI	• Average of five continuous assessment tests	A positive significant correlation was found between the Attention to Feelings subscale and academic performance, while total EI, the Clarity subscale, and the Mood Repair subscale were not significantly associated with academic performance
Nath, Ghosh, and Das [38*]	India	EQ Questionnaire	Trait-based EI	• MBBS examination	No significant correlation was found between of EI and academic performance
Holman et al. [39*]	United States	SSEIT	Trait-based EI	• Final written examination score • Final practical score	No significant correlation was found between EI and academic performance
Aithal et al. [40*]	India	TEIQue	Trait-based EI	• MBBS examination	There was a significant positive correlation between EI and academic performance
Johar, Ehsan, and Khan [41*]	Pakistan	Quick Emotional Intelligence Self-Assessment Questionnaire	Trait-based EI	• Grades in the professional examination	A positive and a significant correlation was found between the Total EI score as well as the Emotional Awareness and the Social Emotional Awareness subscales and grades in professional examination
Altwijri et al. [5*]	Saudi Arabia	SSEIT	Trait-based EI	• Academic Success Inventory for College Students (ASICS) • GPA	A significant positive association was found between overall EI and ASICS, and between EI and GPA
Gore and Jadhav [42*]	India	Trait Emotional Intelligence Questionnaire	Trait-based EI	• Academic success assessment	There was a significant positive correlation between EI and academic success

*EI* emotional intelligence, *EQ-i* Bar-On Emotional Quotient Inventory [43], *MSCEIT* Mayer–Salovey–Caruso Emotional Intelligence Test [44], *SSEIT* The Schutte Self-Report Emotional Intelligence Test [45], *TEIQue* Trait Emotional Intelligence Questionnaire, *GPA* grade point average, *MBBS* Bachelor of Medicine, Bachelor of Surgery

The next section sheds light on possible sources of inconsistency in the primary research based on reviewing the literature on the association between EI and academic success.

### Sources of inconsistency and the need for a quantitative synthesis

Previous meta-analyses on EI provide some explanations for the inconsistent findings in the primary studies. These include culture or country, age, gender, EI tests, EI subscales, and EI task nature (ability-based vs trait-based assessments; [21, 46, 47]). These factors have been recognized in previous studies that assessed the association between EI and academic success in MD programs (see Table 1). For example, Brannick et al. [33*] reported that there was no significant correlation between a trait-based EI scale (Wong and Lu EI Scale; WLEIS) and academic performance, while an ability-based EI test (i.e., Mayer–Salovey–Caruso Emotional Intelligence Test; MSCEIT) was significantly correlated with academic performance. By contrast, some studies identified a significant relationship between academic performance and some of the EI subscales (e.g., optimism, awareness of emotions, and attention to feelings), no significant correlations were observed between academic performance and other EI subscales (e.g., [16*, 35*]). This study deviates from previous literature in its definition of academic success. While some studies assessed academic success using students’ grade point average (GPA; [30*, 31*, 33*]), others used unit/achievement tests (e.g., Austin et al. [23*]; Chew, Zain, and Hassan [32*]; Rajasingam et al. [37*]). Therefore, in addition to the advantage of using a multilevel meta-analysis in addressing the nested data (e.g., unit and finaltest scores are nested in/within GPA), the difference in academic performance criteria was included as a possible moderator that could explain the variability in the mean effect. The effect of culture on EI variance has also been recognized [48]. The current study included works that represented 10 countries in four different continents (North America, Europe, Asia, and Australia; see Table 1). Finally, some studies showed a link between EI and age [49, 50]. All the above factors were considered in the current study to possibly explain the variability in the mean effect size.

### Research questions

Based on the above literature review, this study aims to answer the following research questions:

1. What is the nature and magnitude of the relationship between EI and academic success in undergraduate medical programs?
2. Do moderators such as country, age, EI test, EI task nature, EI subscale, and academic performance criteria explain the variability in reported results in previous studies on the relationship between EI and academic success in undergraduate medical programs?

## Methods

### Search strategy

Potential studies were identified by searching the following databases: *ScienceDirect*, *ProQuest Central*, *ProQuest Digital Dissertation*, *Academic Search Complete*, *ERIC*, *Access Medicine*, *Medline*, and *PsycINFO* up to December 2022. The following keywords were searched in the titles and abstracts: (“emotional intelligence”) AND (“medical students” OR “medical school”) AND (“academic success” OR “performance” OR “GPA”). Moreover, the authors reviewed the reference lists of the three systematic reviews conducted on the same topic [17, 25, 26]. This search resulted in locating 180 works. After eliminating duplicates, we obtained 123 items (113 journal articles, 7 reports, 1 magazine, 1 conference material, and 1 dissertation).

### Selection process

**Fig. 1:**
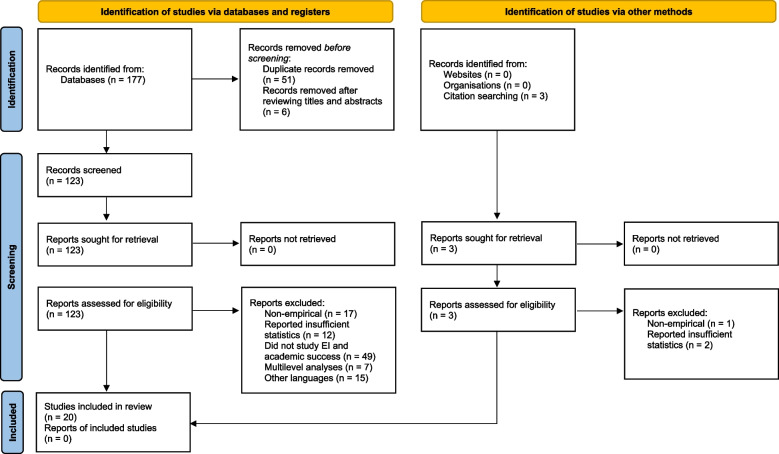
Flowchart for selection of studies

Research studies were selected according to PRISMA guidelines [51]. Five criteria were applied to these 123 works: First, only articles written in English were included. Second, they must report sufficient statistics to calculate the effect size (Pearson’s *r*). Third, they must examine the association between EI as assessed by several well-known EI tests and scales *and* academic success/achievement defined in terms of GPA or achievement examination(s). Fourth, the search included both published and unpublished works; however, only one dissertation was found, which was excluded because it assessed the relationship between EI and leadership [52]. Finally, we only included studies that were conducted with undergraduate medical students pursuing their MD program. All studies conducted on graduate medical students or nursing students were excluded [53, 54]. Applying these criteria brought the number down to 20 studies published between January 2005^1^ to December 2021 (see Fig. 1).

### Data collection

A *coding book*, which included information about the study variables and the special code for each level of categorical variables, was created (see Table 2). The first and second authors met to discuss the coding and clarify any issues before starting the independent coding in the *coding sheet*. In addition to coding the study moderators, the two coders independently retrieved the effect size (i.e., Pearson correlation) and the sample size associated with it. A column for notes was included for the coders to make any comments. The two-way interclass correlation coefficient was high (*r* = 0.93 [55]). All cases with disagreement were individually revisited and resolved by consensus. The data are available on request from the authors.

**Table 2:** Description of the study moderators

Moderator	Operational definitions
*Age*	Mean age of participants; range 18.5–24.1 years
*Year of Publication*	Ranged from 2005 to December 2021
*Country*	The United States and Canada
	Other (include all other countries)
*Academic Performance Criterion*	
GPA	Grade point average
Unit or final examinations	Achievement examinations on a specific unit or module
*EI Test*	
EQ-i	Bar-On Emotional Quotient Inventory
MSCEIT	Mayer–Salovey–Caruso Emotional Intelligence Test
SSEIT	Schutte Self-Report Emotional Intelligence Test
Other EI test	All other EI tests
*EI task nature*	
Self-report/trait-based	A self-report EI assessment that is based on a mixed-model approach for assessing EI
Ability-based	Assessments that treat EI as a set of skills that combines cognition and emotions
*EI subscale*	
Perceiving emotions	Refers to the ability to perceive, control, and evaluate emotions
Emotional management	Refers to the ability to be aware of and constructively handle both positive and challenging emotions
Understanding emotions	Refers to the ability to understand the nature, causes, and control/regulation of emotion
Facilitating thinking	Refers to the ability to use emotions to facilitate thinking
Other	All other EI subscales such as self-expression, stress management, and self-perception
Total score	A composite EI score

### Effect size calculation and statistical analyses

All included studies reported the Pearson product-moment correlation coefficient (*r*). As the Pearson correlation is not normally distributed, each effect size was converted to Fisher’s *z* (see Borenstein and Hedges [56] for transformation equations/formulas).

As most of the studies reported more than one effect size (e.g., an effect size per EI subscale or for each EI test), a three-level meta-analysis approach was adopted, which also considered the various assessments of academic performance. Level 1 referred to the sampling error, Level 2 referred to the between-studies variance, and Level 3 referred to the across-studies variance. All multilevel analyses were conducted using SAS® Studio. The full codes for running analyses can be found in Van den Noortgate et al. [57]. The equations for the full model (without adding moderators) and the full model (where all moderators are included) can be found in Konstantopoulos [58].

### Heterogeneity analysis

There are several methods for estimating heterogeneity in meta-analyses including *Q*, *I* ^2^, and *T* ^2^ statistics. In the current study, both *Q* and *I* ^2^ statistics were computed. The *Q*-statistic follows a chi-squared distribution with degrees of freedom equal to the number of effect sizes/studies minus 1, and it is defined as “the sum of squared deviations of each observed effect from the mean effect on a standardized scale” [59]. *I* ^2^-statistic refers to the proportion of the observed variance. The equations for calculating the Q- and *I* ^2^-statistic are presented in Borenstein et al. [59].

### Assessing publication bias

Three methods of assessing publication bias were used in this study: the funnel plot, Egger’s test, and the Begg and Mazumdar correlation test. The funnel plot is a visual representation, which assumes that in the absence of publication bias, the mean effect size is expected to be the same in small and in large studies [60]. Egger’s test is a parametric test that assesses the funnel plot asymmetry based on linear regression analysis. A significant *t*-test result indicates that publication bias may exist. Finally, Begg and Mazumdar’s test is a nonparametric correlation test that assesses whether there is a relationship between the study size and effect size [61].

## Results

**Fig. 2:**
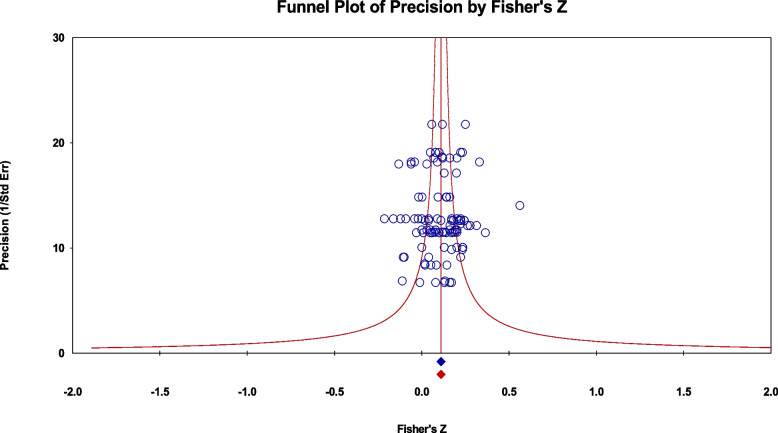
Funnel plot of precision by Fisher’s z

Figure 2 shows the funnel plot for precision. Egger’s regression test was not significant, *b* = -0.59, *SE* = 0.67, *p* = 0.18. In addition, Begg and Mazumdar’s correlation test was not significant, *τ* = -0.02, *z*_*τ*_ = 0.41, *p* = 0.34. These results show that publication bias did not affect the results.

The effect size values ranged between -0.21 and 0.51. To estimate the mean effect size, results from 20 studies (*m* = 105; *N* = 4,227) indicated that, overall, there is a significant positive correlation between EI and academic success, *r* = 0.13, 95% CI [0.08, – 0.27], *p* < 0.01. The within-study variance (Level-2) as well as the between-study variance (Level-3) were both statistically significant (Level-2 = 0.005, *SE* = 0.002, *z* = 1.70, *p* = 0.04; Level-3 = 0.006, *SE* = 0.001, *z* = 3.29, *p* < 0.001). Level 3 explained 29.5% of the variability in the mean effect, whereas Level 2 explained 33.5%. Together, Levels 2 and 3 explained 63% of the variability in the mean effect. As expected, a high heterogeneity was observed, *Q*(105) = 375.48, *p* < 0.001, *I* ^2^ = 72.04.

**Fig. 3:**
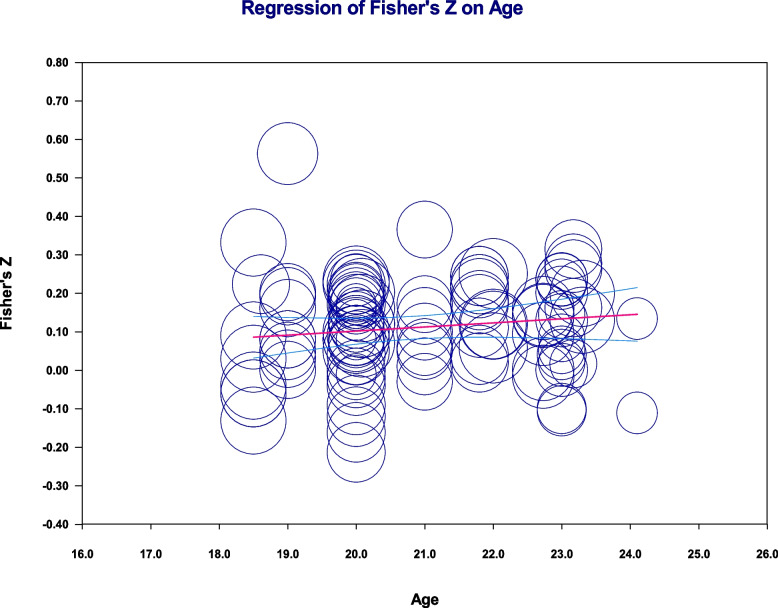
Regression of Fisher’s z on age

Moderator analysis showed that the mean effect size significantly varied according to the EI test, *Q*(3) = 42.93, *p* < 0.001, and EI subscale, *Q*(3) = 18.87, *p* = 0.04, whereas EI task nature [*Q*(1) = 0.71, *p* = 0.40], country [*Q*(1) = 3.08, *p* = 0.08], and academic performance criterion [*Q*(1) = 0.38, *p* = 0.54] did not significantly explain variability in the mean effect (Table 3). The EI test and EI subscale explained 34% of the variability in the mean effect. Age was treated as a continuous variable, and the results showed that age did not significantly explain variability in the mean effect, *b* = 0.011, *SE* = 0.007, *p* = 0.17 (see Fig. 3). As Table 3 shows, the EQ-i test was highly correlated with academic success compared with other EI tests, and the perceiving emotions subscale was highly associated with academic performance compared with other EI subscales.

**Table 3:** Effect sizes by each level of moderators and variance components

Moderator	*m*	*r*	*95% CI*	*p*	*Q*	*I*^*2*^
*EI task nature*						
Ability-based	68	.11	[.074–.148]	< .001	276.38	75.76%
Trait-based	38	.08	[.048–.127]	< .001	97.27	61.96%
*EI test*						
EQ-i	11	.14	[.076–.204]	< .001	20.74	51.79%
MSCEIT	53	.10	[.053–.140]	< .001	210.26	75.27%
SSEIT	6	.11	[-.053–.237]	.109	.995	< 1%
Other	36	.09	[.049–.124]	< .001	99.56	64.84%
*EI subscale*						
Facilitating emotions	8	.13	[.046–.215]	.003	3.21	< 1%
Perceiving emotions	7	.20	[.117–.286]	< .001	.605	< 1%
Understanding emotions	8	.11	[.048–.167]	< .001	.356	< 1%
Managing emotions	8	-.04	[-.238–.163]	.713	107.20	93.47%
Total score	54	.13	[.094–.166]	< .001	159.86	66.85%
Other EI dimensions	21	.05	[-.005–.112]	.071	76.79	73.96%
*Academic performance*						
GPA	37	.09	[.039–.141]	< .001	180.62	80.06%
Examinations	69	.11	[.077–.141]	< .001	194.05	64.96%
*Country*						
US & Canada	48	.07	[.037–.113]	< .001	125.83	62.65%
Other Countries	58	.12	[.085–.161]	< .001	231.06	75.33%
**Variance component**						
Level 2 = 33.5%						
Level 3 = 29.5%						

*EI* emotional intelligence, *EQ-i* Bar-On Emotional Quotient Inventory [43], *MSCEIT* Mayer–Salovey–Caruso Emotional Intelligence Test [44], *SSEIT* The Schutte Self-Report Emotional Intelligence Test [45], *GPA* grade point average; *m* = number of effect sizes; Level-2 = within-studies variance; Level-3 = within-studies variance

## Discussion

The first objective of the current meta-analysis was to test whether EI predicts academic success in MD programs. The three-level multilevel analysis showed that EI is weakly related to academic success in MD programs (*r* = 0.13; 62). It is interesting and surprising that many studies were devoted to investigating such an association. In fact, to the best of our knowledge, there are no MD programs that explicitly teach medical students how to be emotionally intelligent. Therefore, the assumption that academic performance as assessed by different achievement tests in preclinical and clinical years is related to perceiving emotions, understanding emotions, emotional management, and other EI skills might be unrealistic and not based on a solid rationale. To summarize, these pre-clerkship phase tests of the MD program have very little, if anything, to do with EI. Nevertheless, such an interest in EI in medical training has implications. Although EI represents an important skill or set of skills crucial for all careers/jobs, it is especially relevant in health professions wherein many doctors while dealing with patients require EI for optimal health care delivery. Even those who do not often deal with such challenges require EI skills. We tend to prefer doctors who understand us, show empathy, reduce anxiety, and help us stay optimistic. Therefore, although our findings are based on correlational analysis and do not allow us to draw inferences based on the current study’s findings, we highly recommend that EI skills be explicitly embedded in the MD competency framework. The EI learning outcomes could be achieved either by infusing EI skills into the curriculum or through special training modules. Such a recommendation is supported by Cherry et al. [62] who conducted a critical review of EI in medical education and concluded that “EI-based education may be able to contribute to the teaching of professionalism and communication skills in medicine” (p. 468). Roth et al. discusses practical methods for teaching EI in medical education [63].

A distinction must be made between trait and behavioral EI models because such a distinction has implications on the interpretation of grades achieved in academic courses versus those achieved in clinical settings. While trait EI model predicted the performance of dental graduate students in the Dental Admission Test (DAT) in the first two years in classroom-based didactic courses, the behavioral model of EI predicted grades in the third- and fourth-year clinic-based assessment by dental faculty. Conversely, DAT, the dental school equivalent of MCAT, did not predict grades in the third and fourth years, and behavioral EI did not predict grades in the first two years [64]. Therefore, any interpretation of EI and academic grades of learners, more so in health professions, must consider the EI model used. Nevertheless, the relationship between EI and performance is likely more relevant to the behavioral, interpersonal, and professional aspects of performance than the academic or technical aspects of performance.

Antagonistic neural networks underly differentiated leadership roles in medicine [65]. Analytic processes, including problem solving, emanate from a dominant neural network called the Task Positive Network. However, human interactive processes and openness to new ideas and emotions emanate from the Default Mode Network. These two networks are antagonistic [66]. Given these neural underpinnings, academic grades are not a suitable performance measure of EI in medical school or in the practice of medicine. In addition, there are cross-cultural differences in the EI- scores of medical students [67–69].

The second objective of the current study was to test whether the mean effect size varied based on several moderators (see Table 2). Moderator analyses showed that EI tests and EI subscales significantly explained 34% of the variability in the mean effect. Specifically, EI and academic performance are highly related when a trait-based self-report assessment (i.e., EQ-i) is being used compared with an ability-based EI test (i.e., MSCEIT and Schutte Self-Report Emotional Intelligence Test. This is in line with previous studies that showed a weak correlation between ability-based EI tests and trait-based EI scales [21, 70, 71]. Moreover, according to O’Connor et al. [72] “People are not always good judges of their emotion-related abilities and tendencies” (p. 4). Another general disadvantage of self-report assessments is their susceptibility reporting untrue information. However, the correlation between the EQ-i test and academic performance was still weak (*r* = 0.14). Finally, moderator analysis showed that the EI subscale moderator significantly explained some of the variability in the mean effect. The highest correlation was found for skill in perceiving emotions (*r* = 0.20) followed by facilitating emotions (*r* = 0.13) and understanding emotions (*r* = 0.11). Such a finding indicates that when the ability-based EI test is used with undergraduate medical students (more specifically, the MSCEIT), perceiving emotions best predicts academic performance compared with the other MSCEIT branches.

An important aspect to be considered while interpreting EI in predicting academic success in medical school pertains to the evaluation methods employed to measure academic success. EI affects the major competencies expected of graduating doctors, such as communication skills and professionalism. While there are only a few quality outcomes of these measures, possible outcomes include Objective Structured Clinical Evaluation, faculty ratings, and disciplinary action records. The complexity of evaluating these outcomes may require a 360-degree approach to capture an appropriate level of mastery. Notably, the World Federation of Medical Education as well as MD accreditation commissions have explicitly stated standards and indicators to evaluate these domains. Future studies may focus on these issues to further refine the concept of success in medical school that translates into professional success in medical practice as well.

Two limitations are worth mentioning regarding the current study. First, although performing a meta-analysis study with approximately 15 studies is acceptable [73], researchers in the current study were limited by the small number of effect sizes for some levels of moderators. As Table 3 shows, some levels of moderators consisted of less than 10 effect sizes, which might limit the generalizability of some findings. Second, owing to language limitations, only studies published in English were included. Our search showed that 15 related studies that were published in other languages, which we could not include in our study owing to language limitations.

## Conclusions

We conclude with the following recommendations: (a) As our findings showed that EI and academic success are weakly related, researchers and practitioners in the medical field may want to include EI related skills into the MD curriculum or to target EI skills through professional development training/programs; (b) medical education researchers may shift their focus from correlational to experimental studies where EI is explicitly targeted [62] to ensure that MD graduate have the skills to understand others’ emotions and feelings, show empathy to their patients, control, evaluate, and manage their emotions, and use other EI skills effectively in healthcare delivery.

## Data Availability

Data available upon request from the first author.

## Abbreviations

EI: Emotional intelligence
MD: Doctor of Medicine
MSCEIT: Mayer–Salovey–Caruso Emotional Intelligence Test
TMMS: Trait Meta Mood Scale (TMMS)
CPX: Comprehensive clinical performance
PE: Physical examination
DAT: Dental Admission Test
GPA: Grade point average
UMAT: Undergraduate Medicine and Health Sciences Admission Test
TER: Tertiary Entrance Rank
WLEIS: Wong and Law Emotional Intelligence Scale
STEU: Situational Test of Emotional Understanding
STEM: Situational Test of Emotion Management
ASICS: Academic Success Inventory for College Students
EQ-i: Bar-On Emotional Quotient Inventory
TEIQue: Trait Emotional Intelligence Questionnaire
CGPA: Cumulative grade point average
MBBS: Bachelor of Medicine, Bachelor of Surgery

## Footnotes

1 This is the date of the first published study that met the inclusion criteria. There was no restriction for the year of publication.
